# Where's the CASE (Patient) Today?

**DOI:** 10.4103/0970-0218.42043

**Published:** 2008-07

**Authors:** Viren Kaul

**Affiliations:** Ex-Secretary, IMA Students' Wing (Hubli City), Final Year, Karnataka Institute of Medical Sciences, Hubli, Karnataka, India

## Clinics Ahoy!

It is their first time in the hospital and most of the second years are scared of being questioned, and if they will be able to face the patients. But, at the same time, smiles dance on their lips, for they know that they are on the way to success. Having been assigned to their respective units, they embark on the most important mission of their career, that is, to perfect the art of history taking. Most of the students read their clinical manuals sincerely. Unfortunately, students skip or at most skim through the very first chapter in their books – The art of being a good clinician. Here are sown the seeds of disregard for the patient's privileges.

## What It Takes

It is a doctor's duty and for most, a privilege to help the patient and put him at ease. It does not, always, require mind-numbingly tough surgeries or expensive medicines. Simple courtesies such as warmly shaking their hands, providing a patient hearing, being sensitive to their emotional states helps in establishing a patient-doctor relationship. Furthermore, explaining why they are being put through the agony of being questioned repeatedly, informing about the procedure they are going to be subjected to, and many such others gestures are a part of what doctors can do to make their patients feel better.

## WE, The Students!

Students overlook these general protocols and courtesies and it is usual to see all the 8 or 10 students in a unit firing salvos at the patient, trying to simultaneously palpate or auscultate, or discussing right across the bed. The patients are interrogated in an insensitive manner. When this happens, most of the patients are scared of their wits because they feel that they are suffering from some serious ailment that requires the attention of many physicians. This is only exacerbated when the students, without rendering much information or sympathy, go on buzzing with conversation, ignoring the patients. To add to the mental agony, the books are kept open on the patients' beds and more often than not, the photographs and diagrams in the book are kept very much visible to the patient. This alarms the patient no end since most of the photos shown are vivid.

## WHY Are We So?

The question now arises, why do the students behave in such a manner? It may partly be because of the heavy course load, the rushed schedules, the uncooperative attitude of the patients, or simply because the setup in developing countries fosters such callous attitudes. Government Hospitals in India do a great service in providing world-class treatment at literally no cost. With 23% of the population below poverty line, it is a boon for the common man in India. But just because the patients are being treated at subsidized rates, is it fair to subject them to such uncivil treatment? The patient is expected to keep silently agreeing to their demands and if at all he/she is unable to be of assistance at the whim, he/she is branded as “uncooperative”.

Some students do feel bad for the appalling condition of the patients. And, if they mention it to their colleagues, all they are greeted with are subtle taunts about being a “softie”. Others explain their harsh behavior on the ground that doctors need to be mentally strong and not get emotionally involved in the patient's troubles, for it “makes you weak”. Few others make an excuse that the case needs to be presented at a short notice to their professors and thus they need to be quick and efficient. Not so uncommonly, students just imbibe the attitude from watching their seniors and batch-mates, and thus the very thought of being nice never crosses their minds.(1) On the whole, the consensus puts the reason for this demeanor to be the fact that the students lack formal training (in communication and personality skills) before exposure to patient.

## Please Shake My Hand(2)

Most patients want their doctor to shake hands with them at the very first meeting.(2) A study showed that patients were of the opinion that the doctors should be communicative, compassionate and should attend to psychological and contextual determinants of the illness in addition to biomedical aspects.(3) The patients enter the hospital with such expectations and what they get is a cold-hearted indifference. As a result, patients suffer endlessly and this causes them to lose faith in the whole setup. In the annals of medical ethics, this brand of discourteous conduct has been stated to be a “sin”, part of the seven sins of medicine which include – obscurity, cruelty, bad manners, over-specialization, love of the rare, common stupidity and sloth, by Richard Asher.(4)

While debates rage far and wide as to if medical students deserve introduction as “student doctors”,(5) the question that needs to be asked is – do medical students today deserve this title? Before having ‘Dr.’ prefixed to their names, students need to understand that there is more to being a doctor than just swinging the stethoscopes. It is not to aggravate someone's agony, but to take the person's burden and let him breathe a sigh of relief. What pleasure is there in being successful and still not having your patients' affection? In the long run, this love and respect only turns into trust that we all expect from our patients. Yet, we do not want to invest in that “relationship”. Is that a fair expectation? After all someone rightly said “You reap what you sow”.

“A clinician is as good as his patient's faith”. Keeping this is mind, it would be infinitesimally better if all students and those involved in honing them, start integrating in their day-to-day deeds. Students should be exposed to interactive classes and informed comprehensively about patients' right and the doctors' duties - so that they know how to be on their best conduct and are able to provide the humanity and care that their “cases” deserve and make them into their “patients”. With that, the students would be on the path to become “ideal doctors”.
